# A Grumble about Anæsthetics

**Published:** 1899-02-04

**Authors:** 


### Hospital Clinics and Medical Progress.

### A GRUMBLE ABOUT ANÆSTHETICS.

### Two Puzzled Practitioners and a Layman.

1st Puzzled Practitioner: What vexes me about the whole affair is that one gets no rule as to ordinary daily practice.

2nd P. P.: But surely you don't think that any one drug or any one apparatus will do for all cases?

1st P. P.: Oh, no! Of course, different sorts of cases—for example, patients of different ages—require dealing with in different ways. But, considering the enormous number of times that anæsthesia is produced in the hospitals of London alone in the course of a single year, it does seem to me rather absurd that no general rules can be arrived at, even if they went no further than fixing the best anæsthetic for healthy adults, and the ones to be used in some half-dozen common conditions, such as bronchitis, emphysema, heart disease, Bright's disease, &c. Even that would be better than the present condition of chaos.

Layman: Rather jolly, that. Make it all automatic. Mention the disease, and the machine does the rest. Capital! But where does your professional discretion come in, and your knowledge of your patient's constitution, and all that sort of thing? I always thought you doctors maintained that you never went by rule-of-thumb.

1st P. P.: My dear friend, you clearly do not understand what a hole we are in about these anæsthetics. If you ask me something about the action of calomel or quinine, or something that I am ordering half a dozen times a day, I will tell you what I know of my own knowledge. But in regard to anæsthetics the experience of a general practitioner tells nothing. For hundreds of cases, nay, for thousands of cases, all goes well, and then comes a catastrophe. The whole experience of the whole life of many a practitioner may not give the number of cases which, according to the law of averages, should give a death; so that while one man, taking the greatest pains, may meet with an accident in his first half-dozen cases, another man, splashing on chloroform in what I should call the most careless way, may go through his whole life without seeing a death, and, what is more, may boast of it, and put it forward as proof that his method is the best.

2nd P. P.: So you see the question of exercising our discretion and appealing to our own experience does not come in. The whole experience of the whole life of many a general practitioner may only lead to pig-headedness in wrong-doing. We are bound to appeal to a larger experience, and so we turn to the hospitals, with their thousands of cases every year, and ask for something like a definite ruling.

1st P. P.: And we don't get it.

Layman: I see. Starting with normal man, if there is such a party, you would like to know what is the safest anæsthetic to give him, and then you would like something like a formal sanction for departing from the general rule in cases which are abnormal in certain given ways.

1st P. P.: Yes, that is what we want. But to understand the condition of muddle in which we are at present you must bear in mind that we have not yet got so far as a ruling as to the best way of giving the drugs recommended. Take chloroform, for example. Suppose the Society of Anæsthetists were to rule that in certain conditions chloroform was the appropriate anæsthetic, what would that mean? In some hands it would mean pouring an unmeasured and quite haphazard quantity of chloroform on a towel, and holding it over the patient's face, or it might mean dropping chloroform one drop at a time on to a flannel mask, or a sponge charged with chloroform might be placed in a box over the mouth, or the vapour of chloroform might be produced in a Junker apparatus and sent up the nostril or through a tube placed in the mouth, or the vapour might be supplied under a flannel mask, or into a face piece like that used for ether, or into a similar mask with a feather rigged up over the air-hole to show which way the breathing is going; or, finally, the chloroform might be given as an ordinary Clover's ether inhaler, but without the bag; so you see the administration of chloroform means a very different thing according to the person by whom it is given.

Layman: But are not all these methods merely different little dodges, all having the same intent?

1st P. P.: Not at all. Each man swears by his own method. The one with the towel holds all forms of apparatus as anathema, while the man who goes in for regulated and measured doses regards what he calls the "slapdash method" as practically next door to homicide. Oh, no! Don't imagine that these things are mere details. There are plenty of men who believe in the safety of chloroform, and plenty who will agree that everything depends upon how it is given. But when you ask them, "How should it be given?" they each offer a different plan. That is where the trouble is.

Layman: This all seems to me very complex. Do you mean to say that there is no one universally recognised rule as to the proper way in which chloroform should be administered?

1st P. P.: No. I am afraid there is not.

Layman: Well, you are a funny set of people. But surely you have some rule as to how much you may give?

1st P. P.: Oh, dear, no! The most popular and probably the most largely used method is just to pour on the chloroform, and let the patient breathe what happens to evaporate.

Layman: Then you cannot tell how much you are giving?

1st P. P.: Not a bit.

Layman: But is there no way of regulating the quantity?

1st P. P.: Yes, it can be done either by the Junker or the Clover, but there is a strong prejudice against all forms of apparatus.

2nd P. P.: I don't think you have shown our friend as yet the full complexity of the problem, for it is not merely a question of how to give chloroform, but whether to give it at all; whether ether is not better, or whether to give a mixture of both.

1st P. P.: There was a time when it seemed as if ether would drive chloroform right out of the field, except for children and for bronchitic old people—notwithstanding that it is, as was said at a recent meeting, "beastly stuff," but now there is such a tendency to revert to chloroform that all is upset again.

2nd P. P.: Yes, there is no doubt that the ether men have had the best time. They at least had one definite method of administration, which, with but slight variation, they all followed. And then, although they had deaths now and again, they did not have that string of deaths that have been attributed to chloroform.

Layman: Then why in the name of common sense do you not all take to ether?

2nd P. P.: Partly because chloroform is so simple, so nice, and because in such thousands of cases it did so well that it seemed impossible to get anything better, and partly because many surgeons prefer chloroform.

Layman: But what has the surgeon to do with it? If you send your man to sleep is not that enough for him?

1st P. P.: Surgeons have their likes and dislikes the same as other people. Some like to hear their patients puffing and blowing into an ether bag; they say they are happy as long as they hear their man breathing, while others like the absolute quietness of chloroform, they like their patients limp and placid, and are worried by every movement, even that of respiration, and an anæsthetist must please the surgeon, or he won't be asked again.

2nd P. P.: After all, a surgeon may often operate more safely when the patient lies still, as he does under chloroform; and we must not forget the risk of the operation in thinking about that of the anæsthetic, So you see what a state we are in. Perhaps, however, we shall find a way out by way of local anæsthesia. There is a big future for eucaine B; and, as it is a synthetic compound, we may get something of the same sort, but even better, invented before long.

Layman: What do you mean by local anæsthesia? Do you mean freezing?

2nd P. P.: No. I mean the injection of eucaine all about the part to be operated on. A pretty complete local insensibility is produced. A good many cases have been done lately, and when we hear of a man smoking a cigarette and drinking tea while having a radical cure for hernia done upon him, and complaining of nothing except that the tea was too hot, as happened lately at one of our great hospitals, we feel that we do not know where the end will be. Before another jubilee of anæsthesia arrives chloroform and ether may be forgotten.

Layman: All the same, I cannot see why you do not come to something practical, and arrive at some conclusion about chloroform and ether and how to give them. From your account, I should say that the experience gained year by year in your hospitals is being frittered away. The fact is, you want a dictator who would order you about. The question seems to lie between ether and chloroform, and, as to chloroform, between two or three ways of administration. Out of something less than half a dozen methods you want to know which is the safest, and you have a dozen big hospitals in London in which to try the experiment. You just want an organiser to arrange the matter. If each hospital worked exclusively on one plan, a couple of years' experience ought to decide the question.

1st P. P.: Not exactly, because it never is likely to be true that any one anæsthetic will be the best for all cases. But such an experiment might go a long way in deciding in regard to healthy people whether ether or chloroform is the safest; and, as to chloroform, whether the open method or that by regulated doses is the best. As things are at present each man becomes so clever in his own method that he is led into error, and at best individual experience is too small to decide the matter.

2nd P. P.: Yes; there is no doubt that your plan might at least settle for good and all the elementary questions. And, after all, does it not seem a little bit absurd to dogmatise about the difficult cases until we can obtain something like a unanimous answer to the simple question, What is the best anæsthetic for a healthy man?

